# A Multi-DoF Prosthetic Hand Finger Joint Controller for Wearable sEMG Sensors by Nonlinear Autoregressive Exogenous Model

**DOI:** 10.3390/s21082576

**Published:** 2021-04-07

**Authors:** Zhaolong Gao, Rongyu Tang, Qiang Huang, Jiping He

**Affiliations:** 1 Key Laboratory of Ministry of Education for Image Processing and Intelligent Control, School of Artificial Intelligence and Automation, Huazhong University of Science and Technology, Wuhan 430074, China; zhaolong_gao@hust.edu.cn; 2 Beijing Advanced Innovation Center for Intelligent Robots and Systems, Beijing Institute of Technology, Beijing 100081, China; qhuang@bit.edu.cn (Q.H.); jiping.he@bit.edu.cn (J.H.)

**Keywords:** EMG, NARX, neural network, prosthesis

## Abstract

The loss of mobility function and sensory information from the arm, hand, and fingertips hampers the activities of daily living (ADL) of patients. A modern bionic prosthetic hand can compensate for the lost functions and realize multiple degree of freedom (DoF) movements. However, the commercially available prosthetic hands usually have limited DoFs due to limited sensors and lack of stable classification algorithms. This study aimed to propose a controller for finger joint angle estimation by surface electromyography (sEMG). The sEMG data used for training were gathered with the Myo armband, which is a commercial EMG sensor. Two features in the time domain were extracted and fed into a nonlinear autoregressive model with exogenous inputs (NARX). The NARX model was trained with pre-selected parameters using the Levenberg–Marquardt algorithm. Comparing with the targets, the regression correlation coefficient (R) of the model outputs was more than 0.982 over all test subjects, and the mean square error was less than 10.02 for a signal range in arbitrary units equal to [0, 255]. The study also demonstrated that the proposed model could be used in daily life movements with good accuracy and generalization abilities.

## 1. Introduction

In the United States, an estimated 1.6 million people live with limb loss, and 50,000–100,000 new amputations occur every year. Around one fourth of the limb amputees have upper-limb amputation [1]. The amputation results in the loss of mobility function and sensory information of the arm, hand, and fingertips, which severely compromises the activities of daily living (ADL) of the amputees. In addition to the high prevalence of amputation, the demand for improved upper extremity prostheses has increased. However, the traditional body-powered prostheses, which rely on a cable system to move, allow only limited degree of freedom (DoF) movement. With the recent development of robotics and bioinformatics, bionic prostheses have shown promising potential for rehabilitation and improving the ADL qualities of amputees. The bionic prostheses, powered by electronic motors, can perform various motions with multiple modes and DoFs. However, most of them are confined to the laboratory environment; the commercially available bionic prostheses usually have only limited numbers of DoFs. This has compromised the use of bionic prostheses and led to a high abandon rate [2]. The limitation of DoF is mainly caused by poor signal quality and controller algorithms. A real-time multiple DoF control method for a commercially available bionic prosthesis still needs exploration.

The inputs of the controller of bionic prostheses are obtained from biological signals of the user and translate into commands of prostheses with the help of a human–machine interface (HMI). HMI enables the amputees to interact with robots and prostheses to recover or compensate for their lost function. For HMIs in bionic prostheses, the applicable biological signals range from biomechanical parameters of the limb to the electrophysiological activities in the neural system. In the last few years, electroencephalogram (EEG), electromyography (EMG), and various types of biological signals have been applied in HMIs [3,4,5]. Collinger et al. proposed a method to control neuro-prosthesis by decoding the activity of the motor cortex neurons [6]. Participants with tetraplegia successfully controlled a prosthetic limb of seven DoFs via brain–machine interfaces, which related the firing rate of the neurons to the movement velocity of a modular prosthetic limb. Smith et al. achieved simultaneous proportional control of a prosthetic wrist and hand with multiple DoFs [7]. The input signals were gathered by intramuscular EMG with dual-site configuration. However, these approaches are invasive and usually involve surgical procedures for electrode implantation. The surgery of implantation limits the usability of invasive HMIs [8,9]. The risk of infectiousness during the process, contingent rejection reaction, potential deterioration of signal qualities, and other medical concerns challenge the acceptance of invasive HMIs. These disadvantages compromise the application of invasive HMIs in daily-use prostheses requiring a presumably long service life. With these considerations, the non-invasive approaches are superior, especially in terms of commercially available extremity prostheses.

**Figure 1:**
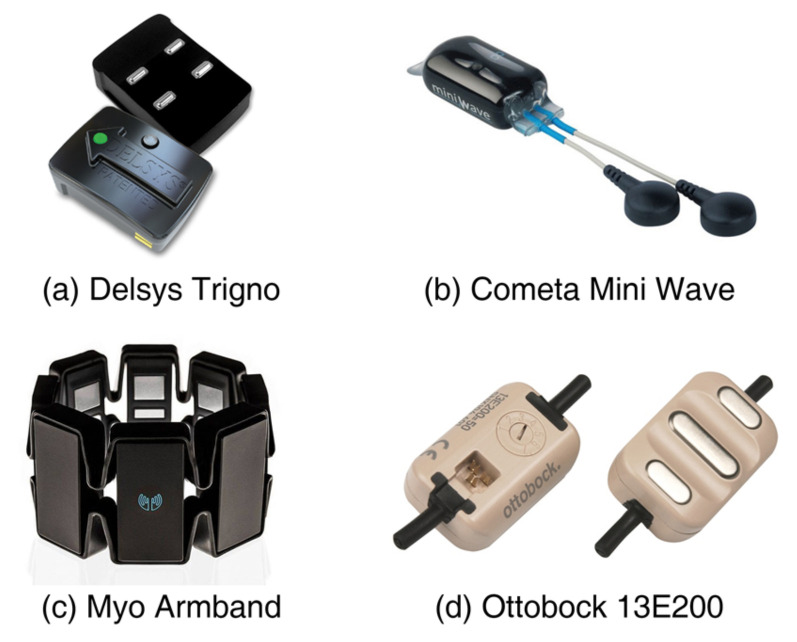
Examples of commercial surface electromyography (sEMG) sensors. (**a**) Delsys Trigno (http://www.delsys.com/, accessed on 25 October 2020). (**b**) Cometa Mini Wave (http://www.cometasystems.com/, accessed on 25 October 2020). (**c**) Myo armband (http://www.thalmic.com/, accessed on 25 January 2018). (**d**) Ottobock 13E200 (http://www.ottobock.com/, accessed on 25 October 2020).

The non-invasive electrophysiological-based HMI uses neural activity signals, such as EEG, EMG, etc., as input signals [10,11]. In particular, surface electromyography (sEMG) is a choice applied with a long history. Surface EMG signals are formed by the variations in states of skeletal muscles, which are triggered by the human intention transferred from neurons to corresponding limbs. The human intention can be reflected directly with no loss and low latency. Therefore, sEMG signals are chosen to be an effective source for prostheses control due to the noninvasiveness and rich information [12]. Compared with other non-invasive approaches such as sonomyography (SMG) [13] and steady-state visual evoked potential [14], the size and weight of the sensors for sEMG acquisition are more suitable for wearable requirements [15] and compact enough for fitting inside a prosthetic hand device [16], as shown in Figure 1. However, this also limits the quality of the acquired signal due to the geometric requirements. To overcome this dilemma, a control system that takes the signal quality of the sEMG sensor under geometric constraints into account needs to be investigated.

For a general control architecture of sEMG-based prostheses, mainly three levels of components are used [17]: top-level, mid-level, and low-level controllers. The top-level controller concentrates on interpreting human intention. The mid-level controller translates the human intention into different motion modes. The low-level controller determines the input parameters of the motor of the prosthesis. The three-level architecture links the raw electrophysiological signals to the control signals of the actuator.

**Figure 2:**
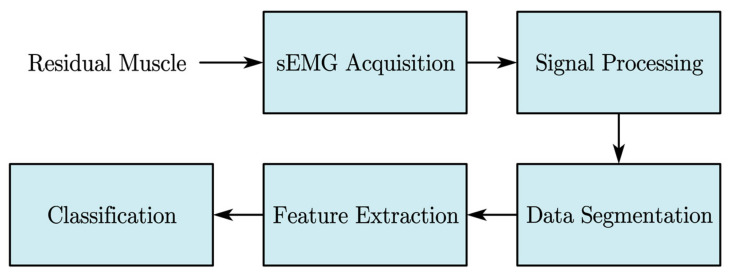
Typical top-level controller of an sEMG-based prosthesis.

In commercially available prostheses, the top-level controller consists of several components, as shown in Figure 2. The signal processing module denoises the input signal and is usually accompanied by dimensional reduction. The feature extraction module calculates the hand-crafted features for classification, and finally the classifier produces the results from the windowed features for the mid-level controller. Two categories of controllers can be distinguished based on the outputs of the classification module. The first approach uses a processed sEMG signal as a trigger for different movement modes. In this approach, researchers use sEMG signals to recognize limb motion and try to achieve more modes and higher recognition rates. Hence, studies concentrated on feature extraction methods and classification algorithms [18,19,20,21]. However, the mode switching methods are limited by the number of modes available. The predetermined modes of movements cannot reflect the user’s intended motion precisely. As a result, the embodiment of the control and the smoothness of motion are compromised. The second approach is to continuously estimate the motion variables from human intention to achieve smooth control of the prosthetic hand. For instance, many methods attempted to relate the sEMG signals to the joint angles of the prosthetic limb. Lloyd and Besier constructed a forward biomechanical model for joint torque calculation with sEMG signals [22]. The mapping of sEMG signals to joint variables such as torque and angles can also be achieved with polynomial fitting [23] or neural network [24,25,26]. In these methods, the motion control of the prosthesis is continuous allowing more dexterous movements and the trajectory is not confined to pre-set modes. These continuous methods have some limitations. For example, the body parameters are highly user dependent and hence limiting the generalization capability and requiring a large data set for training.

In commercial applications, a variety of sophisticated prostheses choose sEMG as their input signal. While significant improvement has been achieved over the past decades, the controllable DoFs are still limited. For instance, the Otto Bock’s Sensorhand Speed [27] only enforced single-DoF grippers. It was equipped with two sEMG electrodes [28] placed on the loci of the residual muscle with maximum electrophysiological activity. The limited DoFs compromised the use of sEMG-based prosthesis. Meanwhile, the unreliable signals of the wearable sEMG sensors were due to the intrinsic variety of neural activities, electrode shift, sweat, and muscle fatigue [29]. As a result, the abandonment rate of hand prostheses remains high [30,31]. Additionally, the features affected the accuracy of the classification controller significantly. As a result, the feature selection is of vital importance for sEMG-based HMI. However, the hand-crafted features compromise the generalization ability of the HMI. To avoid feature selection, raw sEMG signals should be fed directly into the estimation algorithm, and hence the feature selection and processing can be done inside the controller. Thus, accurate and fast feature selection and extraction for joint angle estimation are still considered a challenging task for commercially available extremity prosthesis.

With the recent advent of multi-DoF hand prostheses, the need for a dexterous multiple DoFs top-level controller for HMI has become urgent. In an ideal controller, every degree of freedom is proportionally controllable by the user intent independently. Many studies have been conducted to augment the performance of sEMG signals in terms of acquisition, denoising, and classification. New advances in classification algorithms have been made with the help of machine learning. Furui et al. [32] employed an sEMG-based motion generator to realize the control of multiple finger movements, with the introduction of advanced control mechanisms based on human motion. Both healthy and upper-limb amputation individuals participated in the evaluation test.

In this study, a nonlinear autoregressive exogenous (NARX)-based model was proposed to estimate finger joint angles which could be used as targets for the prosthetic hand top-level controller. The training and test data used in the experiment were from the 5th NinaPro database which focused on the signals of wearable sEMG sensors suitable for a commercially available prosthetic hand. In this database, a Myo armband was used as the sEMG acquisition device. The Myo armband could work without extra batteries and transfer data wirelessly with adequate sample frequency and accuracy. This database established a platform for sEMG-based controller design. First, the sEMG signals were fed into the preprocessing module, by which the sEMG signals were segmented and their dimension was reduced by applying Principal Component Analysis (PCA). The features were extracted in the time domain. Then, an NARX network was employed to select and map the optimal sEMG features to the pre-chosen space of joint angles. Finally, an inverse PCA and Kalman filter were applied for post-processing to convert the reduced space into the original joint angle space after signal conditioning. The model proposed in this study outperforms the multilayer perceptron neural networks. It demonstrated a good generalization ability to simplify the learning process for users. It aimed to realize the continuous sEMG control in wearable devices for HMIs and enhance the stability in extremity prosthesis.

This manuscript is organized as follow. Section 2.1 presents the details of data acquisition and the preprocessing of the NinaPro database of finger movements used in this study. The background and implementation details of the NARX controller are presented in Section 2.2. The results and discussion are presented in Section 3 followed by the conclusion of this study in Section 4.

## 2. Materials and Methods

### 2.1. NinaPro Database

In this study, the NinaPro database was used for training and validation of the algorithm. The database contained data from different acquisition setups. Among these, the Double Myo dataset (DB5), which used Myo armband (Thalmic Labs, Kitchener, ON, Canada) as the acquisition device, was chosen to simulate the input signal from wearable sEMG sensors of commercial prosthetic hands. The electrode placement and the signal quality of DB5 were similar to those applied in the commercially available prostheses, making the database particularly suitable for the exploration of sEMG recognition in commercially available devices.

#### 2.1.1. Subjects and Acquisition Setups

**Figure 3:**
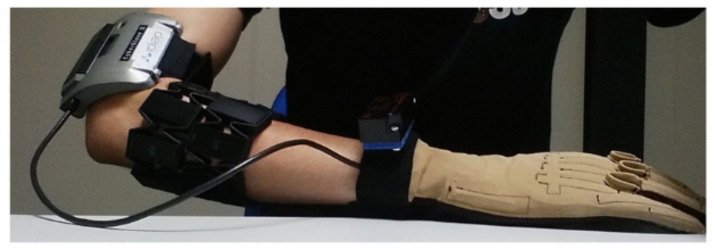
Myo armband and dataglove setup in data acquisition experiments [33] (Adapted with permission license CC BY 4.0 (2017)).

The experiment data of 10 intact participants (8 males and 2 females) were included in DB5. The sEMG signals were recorded with two Myo armbands around the forearm of the participants as shown in Figure 3. There are eight stainless-steel single differential electrodes, which could be evenly placed around the forearm of the user. The placement of the upper armband was closer to the elbow, with the first electrode on the radio humeral joint and the lower one next to the first with 22.5 degrees rotation. The sEMG was sampled by the electrodes of the Myo armband at 200 Hz. The data stream was transferred to the computer via Bluetooth. The configuration of the Double Myo armband was a uniform and affordable platform for muscle exploration and mapping and algorithms of the top-level controller in a commercial prosthetic hand.

**Figure 4:**
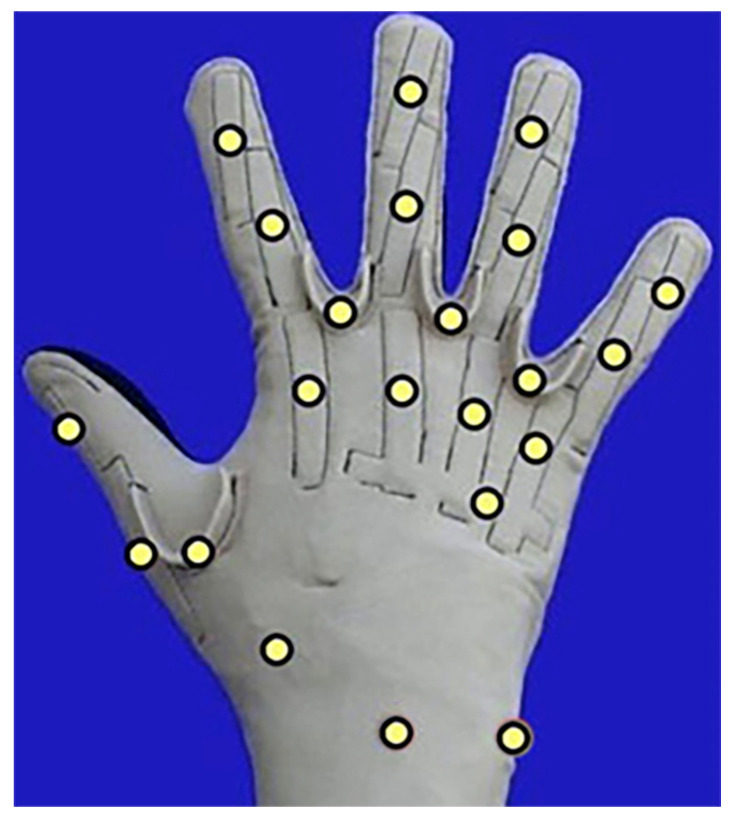
Strain gauge placement of the CyberGlove II.

The kinematic data of the hand were recorded using a CyberGlove II dataglove (CyberGlove Systems LLC, San Jose, CA, USA) with 22 strain gauge sensors. The CyberGlove captured the motion of fingers by measuring the joint angles, as shown in Figure 4. The return value of the strain gauge was proportional to the joint angle with an 8-bit resolution. The data of sEMG signals from Myo armband and the kinematic data from CyberGlove II are all dimensionless. Their range is [0, 255].

#### 2.1.2. Acquisition Protocol

The participants in the DB5 database were requested to perform three kinds of exercises: 52 movements including basic finger movement (Exercise 1), isometric/isotonic hand configurations (Exercise 2), and functional movements in daily life (Exercise 3) [34]. They were asked to follow the movements represented by a screen in front of them.

The movements selected for model training were the signals of Exercise 1 in the protocol. The movements in this exercise were basic movements of flexion and extension of each finger. The details of the movements are described in [34].

### 2.2. NARX Controller

**Figure 5:**
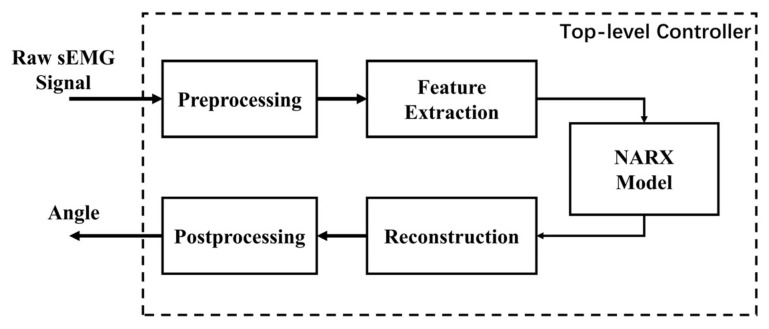
Block diagram of the nonlinear autoregressive exogenous (NARX) model-based top-level controller.

The block diagram of the proposed top-level controller is shown in Figure 5. The raw sEMG data collected from the sensors of the prosthetic hand were sent to the feature extraction module after preprocessing. Then, the features were extracted to construct the input vector for the NARX model. The number of output dimensions of the NARX model was consistent with the DoFs of the prosthetic hand after dimension reduction by PCA. The output of the NARX model was reconstructed into the original dimensions of the controllable joints of the prosthetic hand via inverse PCA. Post-processing was performed to ensure the continuity and robustness of the output target angles in the case of dangerous movements.

#### 2.2.1. Data Preprocessing

The raw sEMG data from the Myo armband and joint angle data from Cyberglove II needed to be processed before NARX model training. The sEMG signal and the joint angle data were merged and synchronized according to the timestamp and resampled to 200 Hz.

**Figure 6:**
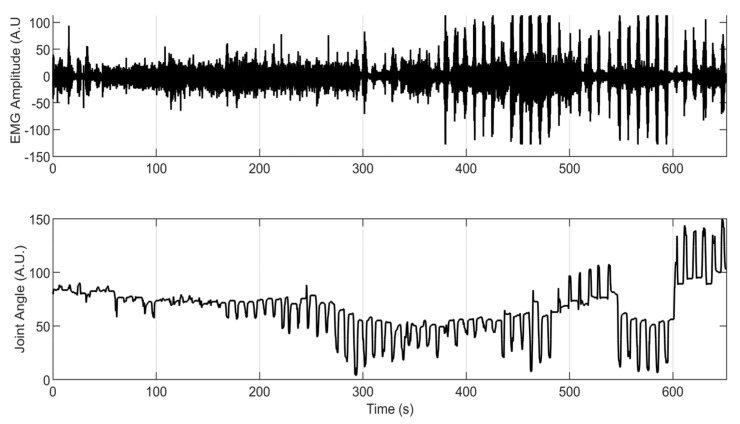
Representative EMG signals and dataglove signals (A.U.: Arbitrary Units).

- Surface EMG Signal: The powerline interference was removed by a 50 Hz notch filter. In NinaPro DB5, the labels of the movements were also recorded along with the sEMG signals. The labels were corrected with algorithms described in [35,36] for synchronization and removal of errors.
- Dataglove Signal: For the CyberGlove joint angle data, PCA [37] was applied to the data for dimension reduction. The first six components with the largest variances, that is, the eigenvalues of the covariance matrix, were chosen as the target controller outputs. The PCA algorithm converted the original dataset into a new space allowing the control of multiple DoFs with limited controller outputs. In this study, the first six PCs were considered significant to the multiple DoF spaces. By employing the “inverse PCA” algorithm, the controller could remap PCs to the original DoFs of the prosthetic hand using the PC matrix obtained from experimental data before generating the final outputs. Thus, all DoFs of a dexterous prosthesis might be controlled in synergy with the constraints of sEMG signals. This method was implemented in the reconstruction module, as shown in Figure 5. The signals after preprocessing are shown in Figure 6.

#### 2.2.2. Feature Extraction

Before feeding into the NARX network, the features needed to be computed from the preprocessed sEMG signals. However, the extraction of user intention from the raw sEMG signal was of high computational complexity due to the intrinsic attributes of sEMG signals, which were stochastic, complex, and nonlinear. This limited the use of sEMG signals in real-time oriented applications [38].

Data segmentation was conducted prior to feature extraction. The time windows were set to 200 ms to avoid high computational costs. The segmentation in this controller was based on non-overlapping sliding windows to enhance the real-time processing ability of the controller. The interval of the sliding window was set to 200 ms to meet the requirement of real-time control with acceptable latency.

The features employed in this application were time-domain statistical values. A lot of them could be used as features for the classification of hand movement. The features chosen in this study were root mean square (RMS) and zero crossing (ZC). The equation for RMS and ZC are given as (1) and (2), separately, and the details are described in [39].

(1) $$\mathrm{RMS}=\sqrt{\frac{1}{n}\overset{N}{\underset{k=1}{\sum }}x_{k}^{2}}$$

(2) $$\mathrm{ZC}=\overset{N}{\underset{k=1}{\sum }}sign\left(x_{k}x_{k+1}\right)\;and\;\left|x_{k}x_{k+1}\right|\;\geq threshold\;value$$

Due to the limitation of the computational complexity, the feature selected for the real-time controller should cover as many aspects of the data characteristics as possible within adequate dimensions. The two features implemented in this study were both in the time domain to obtain a low reaction time and they covered different aspects of characteristics of the data: RMS related to the amplitude in the time domain and the ZC related to the oscillation frequency.

#### 2.2.3. NARX Model

Neural networks are effective for the pattern classification of unstructured static data, which are time-invariant. In temporal pattern recognition, patterns change over time. Traditional feedforward neural networks, however, are not effective for temporally evolving data. Therefore, the alteration of the structure of the neural network should be done to consider temporal dependences during training.

**Figure 7:**
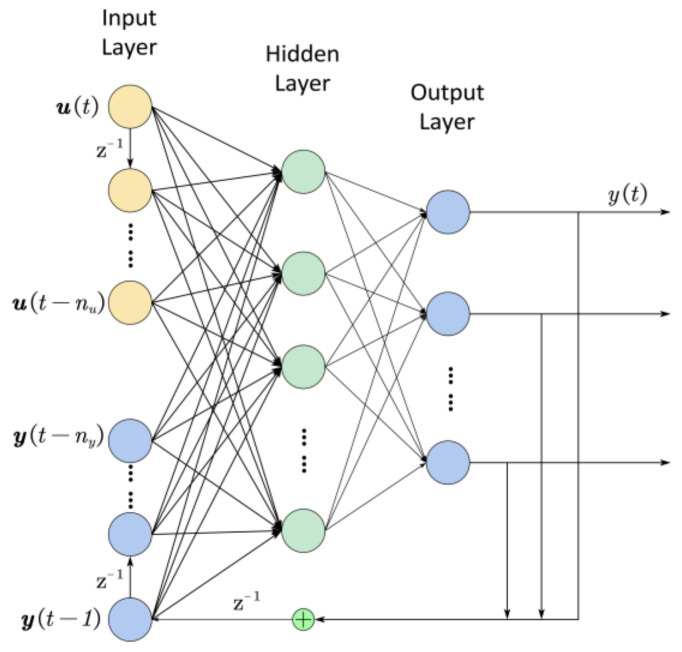
Diagram of the NARX model network structure.

A NARX model was employed in this study for estimating the angular displacement of the fingers. NARX structure-based modeling was developed to capture the nonlinear dynamics of the temporal system [40]. The definition of the model is given by

(3) $$y\left(t\right)=f\left(\omega \left(t\right)\right)$$

and

(4) $$\omega \left(t\right)=\left[\begin{array}{c} u\left(t\right)\\ u\left(t-1\right)\\ \vdots \\ u\left(t-n_{u}\right)\\ y\left(t-1\right)\\ y\left(t-2\right)\\ \vdots \\ y\left(t-n_{y}\right) \end{array}\right]$$

where y(t) is the estimated output with the input u(t) by the regression vector ω(t) at time t. u and y are the input and output vectors of the system, respectively [39]. Figure 7 shows the diagram of the NARX model network structure. NARX model network contained a multilayer feedforward network and a recurrent neural network with time delays [41]. The recurrent neural network, in addition to the feedforward neural network, used the output of the last state as the input of the next state in a feedback loop. The proposed model had three layers: (1) Input layer with delays in which the input layer took the inputs $u\left(t-n_{u}\right)$ and the outputs $y\left(t-n_{y}\right)$ from the past, whose numbers were determined beforehand. (2) Hidden layer for feature selection and processing in which the input features were RMS and ZC, and the outputs were the angular displacements of the finger joints. The number of nodes of the hidden layer was chosen by a parameter sweep experiment, which tested the performance of networks with different numbers of hidden nodes. (3) Output layer, which produced the six components in the reduced joint angle space according to the activation of hidden layer nodes. The outputs were fed back to the input layer in the next state.

#### 2.2.4. Training Procedure

The function f in (3) could be approximated by various methods, such as fuzzy methods or neural networks. In the NARX model, the function f was trained with pre-selected parameters using the Levenberg–Marquardt (LM) algorithm. The RMS and ZC features extracted from the sEMG signals were randomly divided into three parts: training (70% of all data), validation (15% of all data), and testing (15% of all data).

The LM algorithm, modified and improved from Newton’s method, combined the advantages of the convergence speed of the Gauss–Newton method and the stability of the steepest descent algorithm. The weight update equation based on the LM algorithm is given by

(5) $$\omega_{n+1}-\omega_{n}=-{\left[J_{n}^{T}\left(x\right)J_{n}\left(x\right)+\mu I\right]}^{-1}J_{n}^{T}\left(x\right)e_{n}\left(x\right)$$

where J is the Jacobian matrix, e(x) is the training error, and I is the identity matrix. The LM algorithm switched between the Gauss–newton method and steepest descent method based on the value of μ. The steepest decent algorithm was activated when μ was large and the Gauss–Newton method was activated when μ was low.

**Figure 8:**
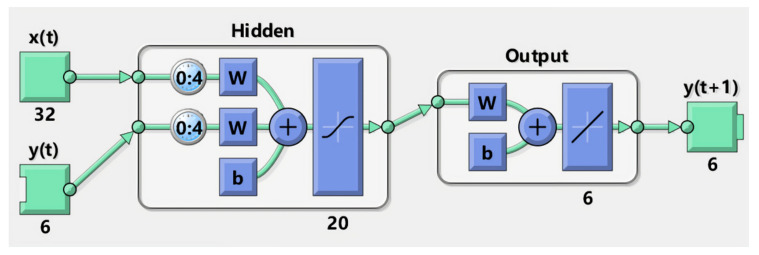
Training setup in MATLAB. The number below each block represents the dimensions. $x\left(t\right)$ contains RMS and zero crossing (ZC) with 16 dimensions of each, and the $y\left(t\right)$ is the output feedback of 6. The delay was set to 4. The value of b in the hidden layer and the output layer was set to 0 initially and updated during the training.

The training NARX model was established using the neural network toolbox in MATLAB (MathWorks, Inc., Natick, CA, USA). The training architecture is shown in Figure 8. The number below each block represents the data dimensions. $x\left(t\right)$ contains RMS and ZC with 16 dimensions of each. They are calculated channel-wise from the 16 channel sEMG signal (8 electrodes times 2 armband). The $y\left(t\right)$ is the output feedback of 6 output values. The delay $n_{u}$ was set to 4. The activation function of the hidden layer was sigmoid function which was defined by

(6) $$\mathrm{sigmoid}\left(x\right)=\frac{1}{1+e^{-x}}$$

The selection of the network parameters and the tuning process were two crucial aspects of achieving optimal performance. The choice of network parameters included the number of neurons and delays. Increasing the number of neurons and delays usually led to an improvement in performance. However, it might also cause the network to over-fit, resulting in low mean square error (MSE) on training data but high MSE on testing data. The network would be considered converged if the performance gradient falls below the pre-selected threshold. Then 6 validation epochs would be performed to ensure the results performance meet the requirement.

#### 2.2.5. Network Parameter Selection

The performance of the neural network model depended on the selection of the input vector and the network parameters. NARX model chose both endogenous and exogenous variables as the input vector, resulting in better performance than using either of them [41]. Since the NARX model was a recurrent neural network, increasing the number of delays in the endogenous inputs and the neurons in the hidden layer usually provided better autocorrelation. However, using a large number of neurons in the network might lead to over-fitting, reducing the performance in out-of-sample data.

**Figure 9:**
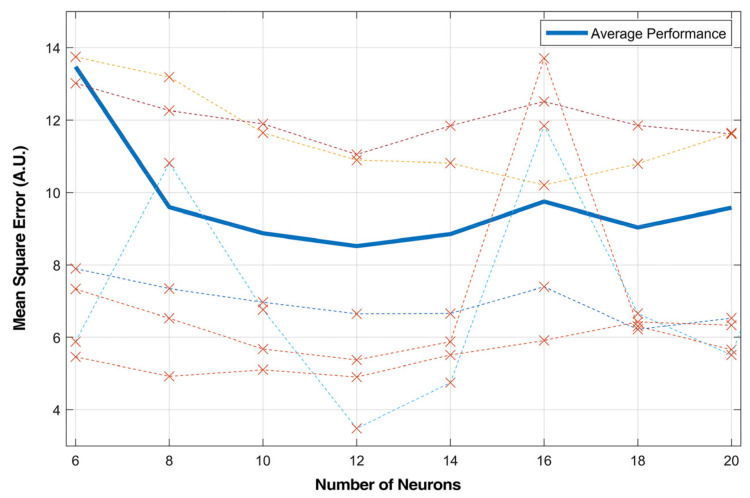
Mean square error with different numbers of neurons. The dashed lines are MSE of different subjects. The average performance was calculated from all subjects.

In the proposed model, the number of hidden layer neurons was swept from 6 to 20 for model evaluation. The results are shown in Figure 9. It proved that increasing the number of neurons in the hidden layer did not necessarily improve the estimation accuracy of a recurrent network. In the parameter sweep test, the best performance was achieved with around 12 neurons. The MSE did not decrease after increasing the number of neurons. Taking the MSE variance over all subjects into consideration, the choice of 14 neurons reached the minimum value, thus increasing the generalization abilities of the model.

**Figure 10:**
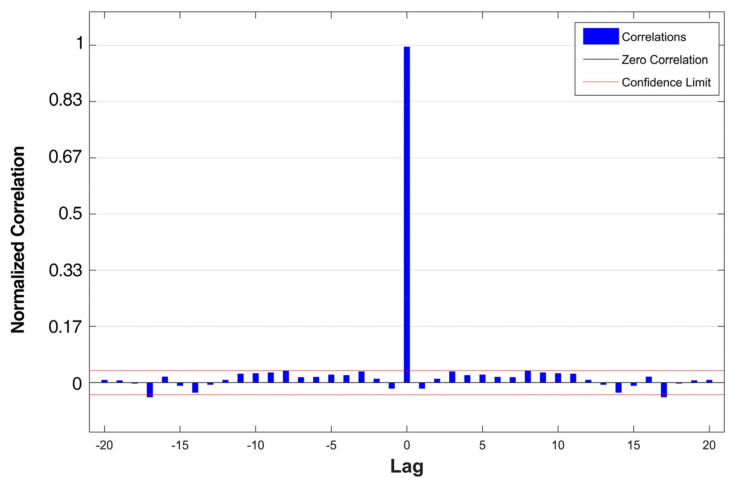
Error autocorrelation of the proposed NARX model.

The error autocorrelation was computed to validate the network after determining the number of hidden neurons. The result of the normalized error autocorrelation function is shown in Figure 10. It was observed that the estimation errors were time related. If the estimation model was perfect, it would be only one nonzero value of autocorrelation occurring at zero lag, indicating that the estimated errors had no correlation. On the contrary, if the estimation errors were significant, the results would imply that improvement of the estimation could probably be obtained by increasing delays or modifying the network structure. In the proposed model, the correlations around the zero lag fell in the 95 percent confidence limits, indicating adequate number of delays in the network. As a comprehensive consideration of various factors, the delay of the proposed NARX model was chosen to be 4 with the corresponding number of neurons set to 14.

#### 2.2.6. Post-Processing

**Figure 11:**
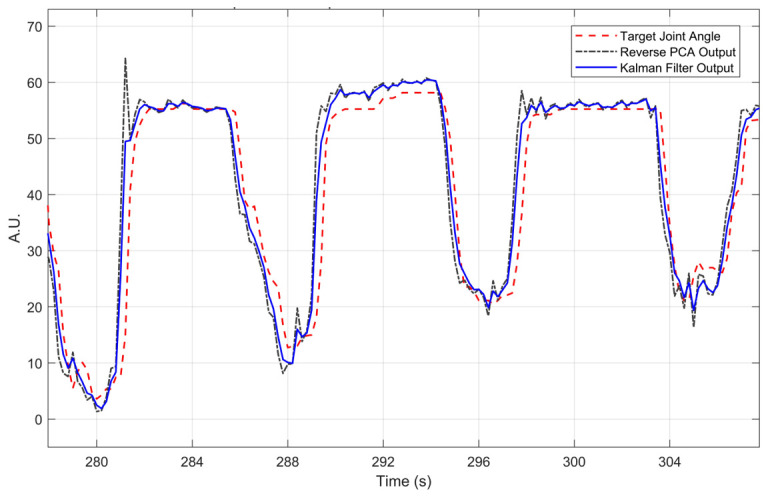
Comparison of output signals after post-processing.

The post-processing module had two steps. First was an inverse PCA converter to reconstruct the signal of the joint angle. The second part was the signal conditioning to improve the accuracy of the output by a simple Extended Kalman Filter (EKF) for the noise canceling. Kalman filters have been applied in time varying systems for better performance [42]. The representative results are shown in Figure 11. The overshoot was reduced by the Kalman filter and the reaction time was not undermined.

## 3. Results and Discussion

### 3.1. Joint Angle Estimation

**Figure 12:**
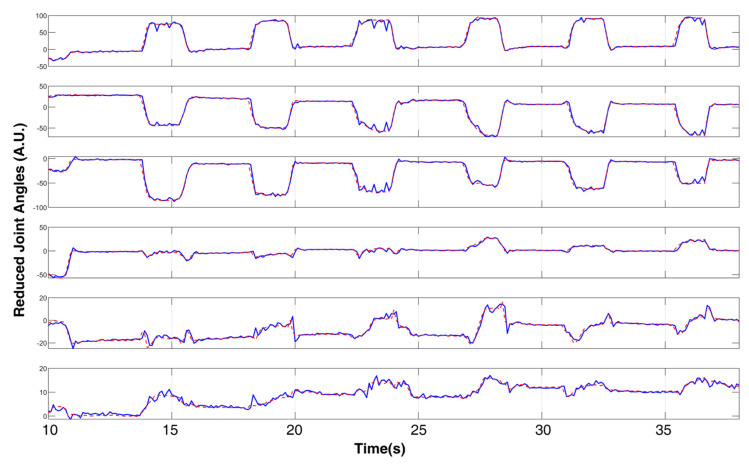
Comparison of NARX outputs and target joint angles in reduced space (all channels).

**Figure 13:**
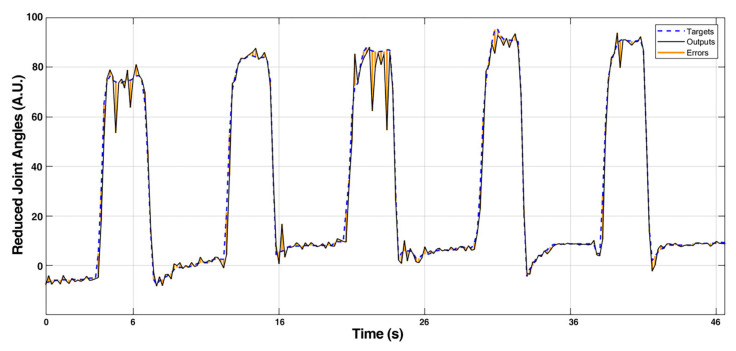
Comparison of NARX outputs and target joint angles in reduced space (single channel).

The example result of the output of the NARX model compared with the target reduced joint angles is shown in Figure 12 (all 6 output channels) and Figure 13 (single channel) from a subject. The output signals in the rest phase were steady, and the reaction time was kept low when the finger movement was triggered. The output signal had good consistency compared with the original target signal. The MSE and regression value of the model were computed to further evaluate the accuracy of the outputs; the results are shown in Table 1. The results indicated that the model was adequate for a multiple DoF application with accuracy and real-time requirements.

**Figure 14:**
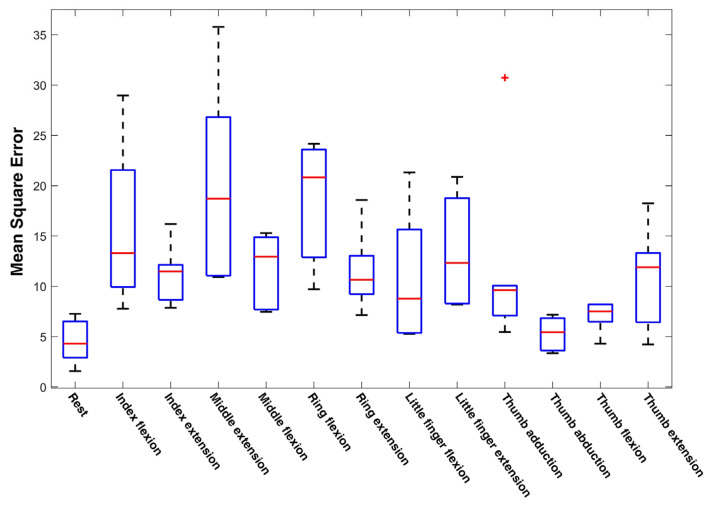
Performance of the proposed model referring to individual movements of fingers.

A comparative study of the proposed NARX model with the traditional multilayer perception neural networks (MLPNN) was conducted. The comparison of the performance of the NARX model with that of MLPNN is shown in Table 2 and Table 3. The comparison was performed over all 10 subjects in NinaPro DB5. The MLPNN, which lacked recurrent layers and delay inputs, was outperformed by NARX in both regression and MSE. The detailed performance referring to individual movements of fingers is shown in Figure 14. All the data of NinaPro DB5 were used for performance evaluation. It is worth noting that the model performed better in extension than flexion implying the differences in the amount of information embedded in the muscle activities.

### 3.2. Generalization Ability

The gathering of training datasets is of vital importance to improve the performance of a neural network model. However, if the algorithm lacks generalization ability, the minimum number of data types for training may be large to cover all possible situations. In this study, the generalization ability of the proposed model was tested by applying the method to hand functional movements in daily life.

**Figure 15:**
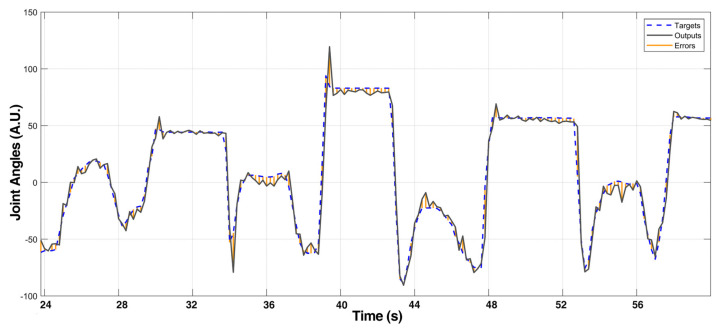
Comparison of NARX model output and the target in daily movements.

**Figure 16:**
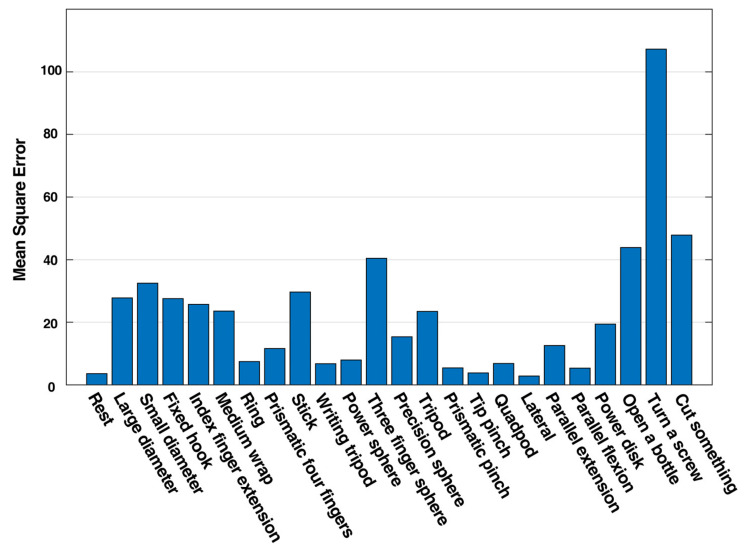
Mean square error of daily functional movements.

In the NinaPro dataset, besides basic finger movements, each participant was required to perform 23 kinds of functional movements in daily activities. In the model training processing, these data were excluded from the training sets on purpose. These datasets were used in this section as the input for the trained NARX network. The comparison of the NARX model output and the target output is shown in Figure 15. The error plot indicates that the maximum error of daily life functional movements samples is less than 13% compared with the target joint angles. The MSE error differed from movements to movements, as shown in Figure 16. It was noteworthy that, approximately, the more the fingers were involved in a single movement the higher the MSE would be. This phenomenon implied that crosstalk might occur between different muscles. The result demonstrated that the pre-training with user data can be done with basic movements solely. Therefore, the user could perform predetermined basic finger movements as the training data for the NARX model controller, which reduced the training process for adoption. The gathering procedure was thus simplified and could be standardized, which was beneficial for reducing the ambiguity in training data. Moreover, the performance could be increased with training data of movements of two fingers simultaneously taking the crosstalk into consideration. As a result, the required time for users to adapt to prosthetic hand movement could be reduced.

## 4. Conclusions

In this study, an NARX-based HMI top-level controller for finger joint angle estimation from sEMG signals was proposed. The sEMG signals used for training were acquired using Myo armband, and the finger movement data were gathered using dataglove. The obtained sEMG and dataglove signals were synchronized and filtered. Two time-domain features (RMS and ZC) of the sEMG signal were extracted and the target data of finger joints were mapped to a reduced dimension using PCA. The time-domain features were then fed to a NARX model trained using the Levenberg–Marquardt algorithm. The result was evaluated with a correlation coefficient of 0.982 and a mean square error of less than 10.02. The comparison of the performance between the NARX model and ordinary MLPNN model was performed. The results indicated that the model was of better accuracy and adequate for a multiple DoF application. The generalization ability of the model was verified with data from daily functional movements, indicating the model can be trained with data from basic movements solely.

## Figures and Tables

**Table 1:** Regression value of the proposed model.

	% of Data	MSE	*R*
Training	70	3.9542	0.989
Validation	15	9.4433	0.985
Testing	15	10.0219	0.982

**Table 2:** MSE of outputs of the NARX model and ordinary multilayer perception neural network (MLPNN) model over subjects.

	NARX	MLPNN
Subject 1	5.687	54.483
Subject 2	10.959	69.872
Subject 3	9.122	62.162
Subject 4	3.881	35.721
Subject 5	5.891	47.635
Subject 6	10.959	76.403
Subject 7	13.027	52.895
Subject 8	32.223	39.113
Subject 9	13.196	47.868
Subject 10	22.843	60.540

**Table 3:** Regression value of outputs of the NARX model and ordinary MLPNN model over subjects.

	NARX	MLPNN
Subject 1	0.9834	0.8054
Subject 2	0.9787	0.7618
Subject 3	0.9797	0.7776
Subject 4	0.9845	0.8187
Subject 5	0.9810	0.7723
Subject 6	0.9791	0.7592
Subject 7	0.9732	0.7930
Subject 8	0.9347	0.7742
Subject 9	0.9762	0.8379
Subject 10	0.9768	0.8787

## Data Availability

Not applicable.
